# A Spatial Prioritization Approach for Identifying Future Conservation Reserve Program Opportunities to Enhance Longleaf Pine (*Pinus palustris*) Ecosystem Connectivity

**DOI:** 10.1007/s00267-026-02593-z

**Published:** 2026-09-03

**Authors:** Benjamin C. Townsend, Nathan P. Nibbelink, Puneet Dwivedi

**Affiliations:** 1 Warnell School of Forestry and Natural Resources, University of Georgia, Athens, GA USA; 2 Department of Forestry and Environmental Conservation, Clemson University, Clemson, SC USA

**Keywords:** Habitat fragmentation, Landscape connectivity, Conservation outcomes, Spatial prioritization

## Abstract

Protecting global biodiversity depends on safeguarding foundation species in forested ecosystems. Longleaf pine (*Pinus palustris*) ecosystems are native to the Southeastern United States. This ecosystem is rich in biodiversity and supports several endemic wildlife species. Over time, longleaf pine has become fragmented. The Conservation Reserve Program (CRP) provides an opportunity to restore longleaf pine in its native range and improve habitat connectivity; however, enrollment is often opportunistic and not necessarily aimed at maximizing environmental benefits at the landscape level, including wildlife habitat. We developed a spatial prioritization approach to identify future CRP opportunities that improve habitat connectivity for longleaf pine-associated species. After identifying CRP-eligible and longleaf-suitable fields, we created resistance layers around each field. Next, we developed two cost surface layers for each field: one incorporating CRP into the landscape and one not. The difference in movement costs between the two scenarios reflected the relative increase in CRP-related connectivity. We identified high uplift opportunities in the Central Florida Ridges and Uplands, the Florida Panhandle, and the Carolina Sandhills. Local landscapes with higher percentages of high-resistance land cover (developed, roads) and higher levels of longleaf pine clustering provided greater uplift in habitat connectivity for CRP opportunities. Our spatial prioritization approach provides information for conservation practitioners that can be adopted or modified into a framework to enhance longleaf pine conservation and the conservation of other foundation species worldwide.

## Introduction

Foundation species are critical for protecting and maintaining global biodiversity, as they shape the conditions that allow other species to thrive in their own native ecosystems. In forested ecosystems, the foundation species is often a tree species (Ellison et al., 2005). As a result of deforestation, foundation tree species have decreased worldwide (Ellison et al., 2005). A consequence of deforestation is habitat fragmentation, in which the spatial structure of forests shifts from large, continuous patches to smaller, isolated patches.

One approach for reducing the effects of habitat fragmentation is to increase habitat connectivity. From a conceptual perspective, habitat connectivity refers to the ease with which wildlife can move through a landscape (Crooks and Sanjayan, 2006). From a modeling perspective, connectivity is often characterized using resistance (or cost) data layers that use land cover and other environmental raster layers to quantify how “resistant” a pixel is to wildlife movement, or how much “cost” is required for wildlife to move through a pixel. These raster layers are then used to calculate pathways between existing patches (Fletcher and Fortin, 2018). One common approach for measuring habitat connectivity and linkages between patches is the least cost path. The least-cost path identifies the path of least resistance for movement between two or more patches (Driezen et al., 2007). Conservation planners have cited habitat connectivity as an effective method to meet a range of wildlife and biodiversity-based conservation goals (Crooks and Sanjayan, 2006; Hohbein and Nibbelink, 2021).

An example of a foundation species whose associated biodiversity is threatened by habitat fragmentation is the longleaf pine (*Pinus palustris*). Historically, the longleaf pine covered large swaths of the southern US, stretching from east Texas to southern Virginia (Peet and Allard, 1993), supporting hundreds of unique plant and animal species, including several longleaf pine specialists (Peet and Allard, 1993). However, in the 19th and early 20th centuries, deforestation for agriculture and over-harvesting greatly diminished the range of longleaf pine, leaving it heavily fragmented across the southeastern US landscape (Peet and Allard, 1993). Habitat fragmentation poses a significant threat to biodiversity conservation in the longleaf pine ecosystem, leading to reduced species richness, abundance, and genetic diversity (Fahrig, 2003).

The Conservation Reserve Program (CRP), a nationwide private lands conservation program, offers an opportunity to restore parts of the longleaf pine ecosystem. CRP is a federally funded program administered by the United States Department of Agriculture (USDA) Farm Service Agency (FSA). Under CRP, farmers agree to adopt a conservation practice on their land, and in exchange, receive annual payments and cost-share assistance to implement it. Contracts last 10 to 15 years, and a field can be enrolled in the program for up to 30 years. Potential conservation practices that landowners can adopt through CRP include establishing wildlife habitats, riparian buffers, and tree plantings (FSA, 2025a). In the southeastern US, many producers have planted longleaf pine on their CRP-enrolled lands in accordance with several regionwide initiatives that are strongly supported by various USDA agencies, including USDA FSA and the USDA Natural Resources Conservation Service (NRCS).

Enhancing and restoring wildlife habitat is one of the most prominently stated goals of CRP (FSA, 2025a). As a result, a substantial body of literature assesses CRP’s effectiveness in improving wildlife habitats. Research on the wildlife benefits of CRP is heavily focused on avian species in Great Plains grasslands, showing that CRP grasslands benefit avian populations positively (Herkert, 2009; Littlefield and Johnson, 2005; Swanson et al., 1999). Other studies, while supporting CRP, suggest that its effects on avian populations may be nuanced. Chief among these caveats is the decreasing effectiveness of CRP for grassland bird abundance and nongame bird nesting habitat over time (Berthelsen and Smith, 1995; Patterson and Best, 1996). In addition to birds, CRP wildlife research includes mammals (Hellesto et al., 2025; Kamler et al., 2005) and invertebrates (Deibert and Utter, 2003; Quinlan et al., 2021). In addition to wildlife, other ecosystem services where CRP’s effect is assessed include water quality (Marton et al., 2013; Schilling and Spooner, 2006), groundwater discharge (Rao and Yang, 2010; Riley et al., 2019), and soil carbon sequestration (Abraha et al., 2018; Li et al., 2017).

Researchers have also used spatial ecology techniques to assess the effects of CRP on wildlife habitat, wildlife home ranges (Kamler et al., 2005; Nagy-Reis et al., 2019) or habitat fragmentation (Adkins et al., 2021; Park and Egbert, 2008). Nagy-Reis et al. (2019) employed multiple spatial scales to assess the importance of CRP for white-tailed deer (Odocoileus *virginianus*) occurrence in North Dakota. They found that at the broad management scale, the percentage of CRP in a hunting unit was one of the most important factors in determining deer abundance. They also found that deer used land cover types associated with CRP land to establish their winter home range, as CRP land provided forage and vegetation cover suitable for fawn bedding. Adkins et al. (2021) used FRAGSTATS to assess greater prairie-chicken (*Tympanuchus cupido*) abundance in Minnesota using three different land cover scenarios based on CRP enrollment patterns. They found that greater prairie-chicken abundance increased most when large and contiguous parcels near existing grasslands and wetlands were enrolled in CRP.

The goal of this study is to identify opportunities in the southeastern US for longleaf pine restoration through CRP to improve habitat connectivity of longleaf pine-associated species. The objectives of our study are to identify environmental and landscape variables that influence increases in habitat connectivity and to test whether a spatial prioritization approach to selecting CRP opportunities for longleaf pine restoration provides greater uplift for habitat connectivity. Our research adds a novel contribution to the existing literature on CRP wildlife in the Southeast US by focusing on habitat connectivity, the longleaf pine ecosystem, and identifying future CRP opportunities. This is especially true as existing research on CRP in the southeastern US has primarily focused on the factors influencing landowner participation in CRP (McLean-Meyinsse et al., 1994; Townsend et al., 2025) and on wildlife (Jones et al., 2009; White et al., 2005). Much of the research on wildlife in the Southeast US focuses on CRP’s influence on improving habitat quality for avian species. White et al. (2005) used measures of landscape composition and configuration to determine how CRP can best be utilized to increase and improve nesting opportunities for northern bobwhites (*Colinus virginianus*). Therefore, our study provides insights into the environmental and spatial dimensions that offer the greatest uplift in habitat connectivity, enabling conservation practitioners to make more informed and cost-effective decisions in the southeastern US, and potentially beyond.

## Methods

### Study Area

The South Atlantic Gulf, a part of the larger southeastern US, spans from central Mississippi to southern Virginia. The South Atlantic Gulf also encompasses the entirety of Florida, and small portions of Louisiana and Tennessee. The South Atlantic Gulf consists of two major ecological zones, the Piedmont, which covers the region’s inland areas, and the Coastal Plain, which runs from the Fall Line to the Atlantic Ocean. The South Atlantic Gulf has a diverse range of land cover types, with mixtures of wetlands, upland forests, riparian forests, and agricultural land (Coffin et al., 2021). The five states that comprise the core of the South Atlantic Gulf (Alabama, Florida, Georgia, North Carolina, and South Carolina) have over 40 million acres of farmland, of which 17 million acres are cropland (NASS, 2024). The South Atlantic Gulf has experienced rapid urbanization and sprawl since the 1950s, especially along the I-85 corridor, which includes major cities such as Atlanta and Charlotte (Terando et al., 2014). Urbanization and urban sprawl are major contributors to habitat fragmentation, and continued urbanization throughout the 21^st^ century is expected to further exacerbate fragmentation in the region (Terando et al., 2014).

### Identifying Opportunities for CRP Longleaf Pine Restoration

The first step was to identify crop fields in the study area eligible for CRP in 2025. We used the USDA National Agricultural Statistics Service (NASS) Crop Sequence Boundaries (CSB). The CSB is a geospatial polygon dataset of crop fields in the contiguous US, delineated algorithmically using the USDA NASS Cropland Data Layer (CDL) to create contiguous fields with similar temporal crop sequences. The CSB contains information on crop land cover for the past 8 years (2017–2024), allowing users to make decisions with field- and sub-field-level information (Hunt et al., 2024). In this study, the term “field” refers to a singular polygon observation in the CSB dataset.

For a crop field to be CRP eligible, a field must be planted in a commodity crop for at least four out of the past 6 years, for the years 2012 through 2017, per the 2018 Farm Bill (FSA, 2026). However, the 2018 Farm Bill expired in September 2023 and has been extended multiple times on a yearly basis until Congress is able to pass a comprehensive Farm Bill (Monke, 2026). When this analysis was started in the fall of 2024, there was an expectation that a comprehensive Farm Bill would be passed in 2025. The 2024 Farm Bill (H.R. 8467, 2024) stated that croplands planted for at least 4 out of the 6 past years before the enactment of the Farm Bill would be CRP eligible. To keep our analysis relevant, we chose the years 2019 through 2024 to be the 6-year time span of CRP eligibility. With the CSB dataset, we used four rules to determine a field’s CRP eligibility: (1) the field was planted in a commodity crop for four or more of the past 6 years, (2) the field was planted in a commodity crop for three or more of the past 5 years, (3) the field was planted in a commodity crop for three or more of the past 4 years, and (4) the field was planted in a commodity crop for each of the 3 past years. Since we are interested in CRP opportunities that could occur in the near future, we use eligibility criteria #2-4 to represent fields that would be CRP eligible in 2025, making the assumption that a field that is a commodity crop in 2024 would once again be a commodity crop in 2025, thereby retaining its CRP eligibility. We followed Spawn-Lee’s (2023) designations of commodity crops.

To determine which CRP eligible fields were the most suitable for longleaf pine, we calculated zonal statistics in R version 4.4.2 (R Core Team, 2024) using the “exact_extract” function in the *exactextractr* package to extract the mean pixel value for two raster datasets that represent landscape-level suitability for longleaf pine (Baston, 2023). The first dataset was LANDFIRE’s 2014 Environmental Site Potential (ESP) data, which maps the best-fitting vegetation type that could occur at a pixel based on its biophysical profile (Rollins, 2009). Using the ESP’s accompanying attribute data, we created a binary raster layer of longleaf pine suitability by reclassifying pixels whose ESP name included the term “longleaf” to a value of “1”, while all other pixels were reclassified to a value of “0”. The second dataset was the Florida Natural Areas Inventory’s (FNAI) 2023 Longleaf Sustainability Analysis (LSA) data, which determines the suitability of a pixel for longleaf pine restoration using Maxent, built on the relationship between existing longleaf pine occurrences and biophysical variables that cover climate, fire history, soils, and vegetation type. The LSA suitability raster ranks a pixel’s suitability for longleaf pine on a scale from 1 to 10, with 10 being the most suitable (FNAI & UF CLCP, 2023). For a field to be considered suitable for longleaf pine, it had to meet two thresholds for zonal statistics, one for each dataset. For the LANDFIRE ESP data, the mean pixel value for the field had to be 1 (all pixels in the field are suitable for longleaf pine). For the FNAI LSA data, the mean pixel value for a field had to be at least 5.

### Identifying CRP Longleaf Pine Opportunities for Habitat Connectivity

For longleaf pine restoration to be most effective for habitat connectivity, there needs to be existing longleaf pine in the surrounding landscape. Therefore, we identified fields where a notable amount of longleaf pine was already present in the local landscape. The first step was to create a raster dataset of existing longleaf pine. The primary dataset used was LANDFIRE’s 2020 Existing Vegetation Type (EVT) data, a land cover dataset that provides detailed species and ecosystem-level information about land cover (Rollins, 2009). Using the EVT’s accompanying attribute data, pixels whose EVT name included the term “longleaf” were reclassified to a value of “1”, and all other pixels were reclassified to a value of “0”. Additionally, we utilized observational data from FNAI’s Southeast Longleaf Pine Database (LEO GDB) through 2025 and existing CRP Longleaf data as of 2024 to fill gaps not present in the EVT dataset (FNAI, 2023).

Once the longleaf pine dataset was created, we placed a 3 km buffer around each CRP-eligible field that was suitable for longleaf pine. A 3 km buffer was selected as the distance to represent an area surrounding a field because it is the average known juvenile dispersal range of species of interest that occupy longleaf pine ecosystems. (Bauder, 2019; Badyaev et al., 1996; Brooks et al., 2019; Cook et al., 2009; Kesler et al., 2010; McRae et al., 1981). Dispersal ranges were identified from the literature for endemic, keystone, threatened, and culturally important species in longleaf pine ecosystems. Table 1 lists the juvenile dispersal ranges of the species used to create the 3 km buffer.

**Table 1:** Juvenile dispersal ranges of the longleaf pine species of interest

Species	Juvenile Dispersal Range (km)	Reference
Gopher Tortoise (*Gopherus polyphemus)*	0.08 km	McRae et al. (1981)
Red-Cockaded Woodpecker (*Dryobates borealis)*	2.94 km (males) 3.31 km (females)	Kesler et al. (2010)
Bobwhite Quail (*Colinus virginianus*)	1.7 km	Cook et al. (2009)
Wild Turkey (*Meleagris gallopavo*)	5.1 km (second year) 6.8 km (third year) 2.6 km (after third year)	Badyaev et al. (1996)
Eastern Indigo Snake (*Drymarchon couperi*)	4.86 km	Bauder (2019)
Reticulated Flatwoods Salamander (*Ambystoma bishopi*)	1.3 km	Brooks et al. (2019)

**Fig. 1:**
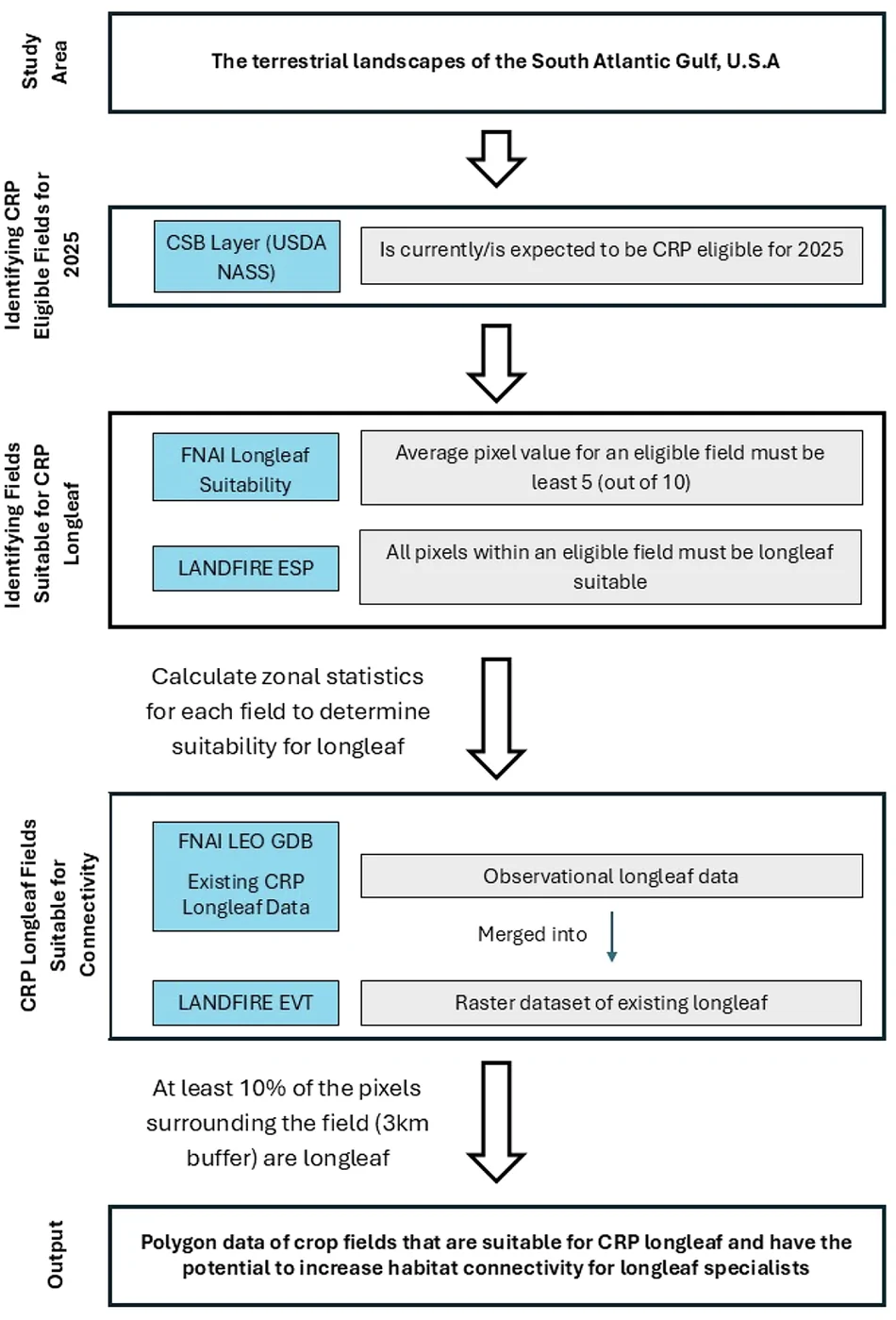
Workflow showing steps taken to identify crop fields eligible and suitable for CRP longleaf pine, and to prioritize CRP fields for improving habitat connectivity for longleaf pine-associated species

Within the 3 km buffer, if at least 10% of the land ( ~ 692 acres/280 ha) was longleaf pine, the field was considered a prime opportunity to improve habitat connectivity, based on the presence of existing longleaf pine stands. We set 10% as the threshold to ensure that sufficient longleaf pine acreage is present in the neighborhood so the region benefits from adding longleaf pine to the landscape. Figure 1 illustrates the entire workflow for identifying CRP longleaf pine opportunities that enhance habitat connectivity. There are a few limitations related to the datasets used to identify potential CRP opportunities. One limitation concerns the LANDFIRE ESP dataset, which assigns the best-fitting vegetation type to each pixel (Rollins, 2009). A consequence of this is that there are pixels, and as a result, agricultural fields, that could be suitable for longleaf pine but were not included in our thresholding due to other vegetation types being a better fit for the land. This means that there may be some high uplift opportunities that were not accounted for. The last data-based limitation concerns the CSB polygons. The CSB fields are delineated from the CDL raster data using an algorithm (Hunt et al., 2024). As a result, some CSB polygons are irregularly shaped, which may not reflect what landowners will enroll in CRP.

### Resistance Layer

The resistance layer was created using the LANDFIRE EVT dataset, supplemented with longleaf pine observational occurrences from existing CRP data and the LEO GDB. In connectivity modeling, a resistance layer is a spatial raster in which each pixel is assigned a value representing the relative cost or difficulty of wildlife movement. Lower resistance values indicate land cover types that are permeable to movement, such as natural vegetation. Higher resistance values represent land cover types that are impermeable to movement, such as roads and developed areas (Fletcher and Fortin, 2018; Hohbein and Nibbelink, 2021). Selecting resistance layer values presents a unique challenge, as true movement costs and wildlife movement preferences are unknown (Bowman et al., 2020). Generally, resistance values themselves (1, 5, 10, etc.) are less important than differences in magnitude between them (Bowman et al., 2020). We selected our resistance values based on resistance classifications of Koen et al. (2014) and Hohbein & Nibbelink (2021). Koen et al. (2014) used values of 10, 100, and 1000, representing low, moderate and high resistances, respectively. Hohbein and Nibbelink (2021) used the same designations, except they added a 500-resistance value to provide greater flexibility. We built upon these designations, adding resistance values of 1 for longleaf pine and 5 for other non-longleaf pine species. This was done to better differentiate focal species’ preferences for longleaf pine and, to a lesser extent, other pine species from those in other natural systems. We separated other pines (not longleaf pine) from other natural land cover types because other native pine species in the south are functionally similar to longleaf pine (Larsen-Gray et al., 2026). Longleaf pine-associated species have been known to inhabit other pines when an open-canopy structure similar to that of longleaf pine exists (Larsen-Gray et al., 2026). Table 2 shows the resistance values assigned to the different land cover types.

**Table 2:** The resistance values assigned to the different land cover types

Land Cover Type	Resistance Value
Longleaf Pine	1
Pine (Non-Longleaf)	5
Natural Vegetation (Non-Pine)	10
Agriculture	100
Roads	500
Developed/Built Areas	1000
No Data	9999

Since there are over 253 land cover classes in the LANDFIRE EVT dataset for the study area, we aggregated them into resistance layers. Resistance values of 1 included any land cover class that had “longleaf pine” in its name, and resistance values of 5 included any non-longleaf pine land cover class that had “pine” in its name. Resistance values of 10 included any non-pine natural vegetation covers. This includes grasslands, broadleaf forests, wetlands, and riparian vegetation. Resistance values of 100 included all land cover classes in the agriculture group, including row crops, pasturelands, fallow, orchards, vineyards, and bushes. Resistance values of 1000 include all land cover classes in the developed group, along with quarries/strip mines and open water. The LANDFIRE EVT data, like any large-scale land cover dataset, can be prone to misclassifications (Puma, 2023). This could result in slight inaccuracies in the cost surface values. However, since our resistance layer aggregates land cover types into six different resistance values, misclassifications should be reduced, as similar land cover types are grouped together.

### Creating Omnidirectional Connectivity Layers

**Fig. 2:**
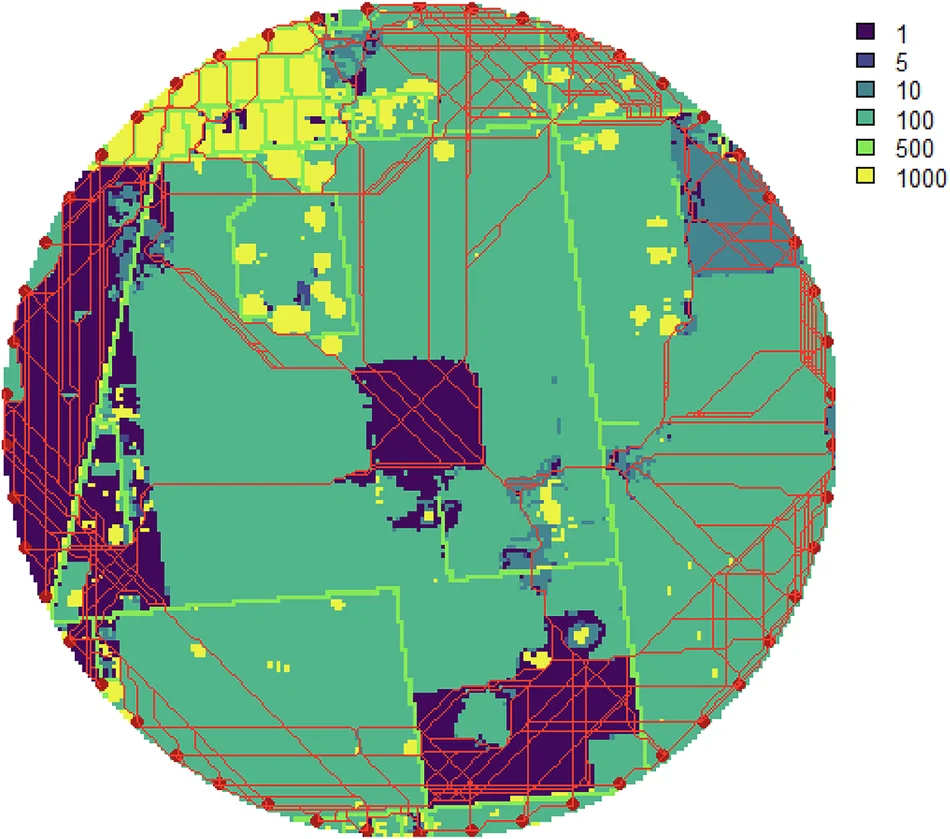
An example of how Omnidirectional connectivity works. The red points on the perimeter represent the nodes. The least cost path is calculated for each possible node pair. The red lines represent the least cost paths from node to node

To calculate the effect of longleaf pine restoration through CRP, we created omnidirectional cost surfaces for each field and its surrounding 3 km buffered landscape. Omnidirectional connectivity measures connectivity throughout a study area from all directions, placing nodes at the perimeter of the study area and calculating connectivity for all possible pairwise node combinations, yielding a dataset that shows the ease or difficulty of movement from any position in the landscape to any other. (Koen et al., 2014; Landau et al., 2021; Phillips et al., 2021). Omnidirectional connectivity is useful in studies where a holistic assessment of connectivity is required, and connectivity between specific target and source patches is not needed (Hohbein and Nibbelink, 2021). To make the cost surface layers omnidirectional, we followed Koen et al. (2014) and placed 50 nodes evenly spaced along a ring around the 3 km buffer around a field. Cost surface layers were calculated for each node pair, yielding 1225 per field. We calculated cost surfaces using the *gdistance* package in R (van Etten, 2017). The first step for calculating cost surfaces was to create a transition matrix of conductance values (the inverse of resistance) for each buffered landscape around the field. The next step was to create the cost surface layers using the “accCost” function in the *gdistance* package. The “accCost” function uses Dijkstra’s algorithm to calculate the shortest path, the “least cost path”, between two nodes (Dijkstra, 1959). Next, we averaged the cost surface layers to create a single cost surface layer representing the mean movement cost for each pixel from any direction. Figure 2 displays the process of omnidirectional connectivity. It is important to note that connectivity models assume a mature longleaf pine landscape, which may not reflect reality. This assumption invites limitations on the effect of our results on the Red Cockaded Woodpecker, which prefers mature (80 years or older) longleaf pine trees to build its cavities, which are the drivers of the Red Cockaded Woodpecker’s keystone species status (Levy et al., 2023).

### Calculating Change in Connectivity

To calculate the effect of CRP longleaf pine restoration on habitat connectivity, we created two different landscape scenarios for each field. The first scenario represents initial landscape resistance conditions, where CRP has not been implemented on the field. The second scenario represents the addition of longleaf pine through CRP, where all pixels that intersect the targeted agricultural field are “converted” to longleaf pine by reassigning their resistance values to 1. Once the two cost surface layers were created for each field, we subtracted the mean pixel value of the pre-CRP cost surface layer from that of the post-CRP cost surface layer. Negative values represent a decrease in movement cost, which can be interpreted as an increase in habitat connectivity. For clarity, we quantified the effect of CRP on longleaf pine as uplift in habitat connectivity (“uplift” hereafter), defined as the absolute value of the change in movement cost. In other words, the greater the numerical value of uplift at a potential CRP site, the greater the potential benefit of that site for habitat connectivity. Since our approach uses the least-cost path method of connectivity, which identifies only the least-resistant movement path, uplift represents the best-case scenario for improving habitat connectivity.

### Analysis

Three different types of analyses were conducted. First, we calculated descriptive statistics for uplift in habitat connectivity across the southeastern US for the following geographies: states, counties, HUC 8 watersheds, and level 4 ecoregions. By calculating descriptive statistics for grouped data, we identified regions of interest for conservation practitioners. In addition, we also identified the distribution of CRP longleaf pine opportunities across species’ ranges used to develop the 3 km buffer. We also included the species range of the Frosted Flatwoods Salamander (*Ambystoma cingulatum)*, a longleaf pine specialist currently listed as federally threatened (USFWS, 2023). Species range data were collected from the USGS’s Gap Analysis Project’s (GAP) Species Range Maps (USGS, 2018). Species range refers to the geographic range of suitable habitat for a species, not the geographic range of current known presence of a species. Bobwhite Quail (*Colinus virginianus*) was not included in the analysis because GAP does not have range data available for it. By considering the number of “species of interest” within a CRP longleaf pine opportunity, we add another dimension to our spatial prioritization workflow and can identify opportunities that may be overlooked because of low uplift in habitat connectivity.

The second analysis aimed to identify potential landscape factors that influence habitat connectivity. This was achieved with two tests. First, correlation was calculated between uplift in habitat connectivity and the following environmental and landscape variables: field acreage, percentage of the 3 km buffer that is longleaf pine, percentage of the 3 km buffer that is high resistance (resistance values of 500 and 1000, representing roads and developed areas), patch cohesion index (PCI) for longleaf pine patches, and the contagion of the landscape. The percentage of the 3 km buffer that is longleaf pine and the percentage of the 3 km buffer that is high resistance are measures of “landscape composition” (Turner and Gardner, 2015). The measures of PCI and contagion are measures of “spatial configuration”, providing insight into the geographic structure of land cover in the 3 km buffer (Turner and Gardner, 2015). PCI measures the connectedness of a land cover type, which is useful for connectivity, and can also be used as a measure of isolation (Schumaker, 1996; Turner and Gardner, 2015). Contagion calculates the probability that a pixel is adjacent (or not adjacent) to a pixel of a different land cover class. Low contagion values represent highly dispersed landscapes, while high contagion values represent highly clustered landscapes. PCI and contagion were calculated using their respective functions (“lsm_c_cohesion” and “lsm_l_contag”) in the *landscapemetrics* package in R (Hesselbarth et al., 2019). The second test ran a linear regression model to assess the relationships between habitat connectivity uplift and the environmental variables (acreage, percent longleaf pine, percent high-resistance areas, PCI, and contagion). Habitat connectivity uplift was the dependent variable, while the environmental variables were the independent variables. Since the distributions of the uplift and acreage values were heavily right-skewed, they were log-transformed to approximate normality. Testing the relationship between changes in movement cost and the above variables provides greater insight into how proximity and proportions of high- and low-resistance values influence connectivity across local landscapes, providing a framework for assessing a landscape’s ability to improve habitat connectivity based on the structure and composition of the existing landscape.

Mann-Whitney U tests were conducted to determine if there was a statistically significant difference between the median uplift in habitat connectivity between potential and existing CRP longleaf pine. Six Mann-Whitney U tests were conducted: five for the following level 4 ecoregions: the Atlantic Southern Loam Plains, the Dougherty Plain, the Carolina Flatwoods, the Sandhills, and the Tallahassee Hills/Valdosta Limesink, along with a Mann-Whitney U test of all five ecoregions combined. The Mann-Whitney U tests enabled us to assess the effectiveness of our spatial prioritization method, as the tests comparing the potential and existing CRP fields demonstrated their efficacy relative to existing selection methods. The FSA uses the Environmental Benefits Index (EBI) to determine which fields to extend CRP offers to. EBI consists of six components: wildlife habitat benefits, water quality benefits, on-farm benefits of reduced erosion, enduring benefits (likelihood practices remain in place after CRP contract), air quality benefits, and cost (FSA, 2025b). Previous research has raised questions about the effectiveness of EBI in measuring the benefits of a field’s enrollment in the CRP (Hellerstein, 2017).

## Results

**Fig. 3:**
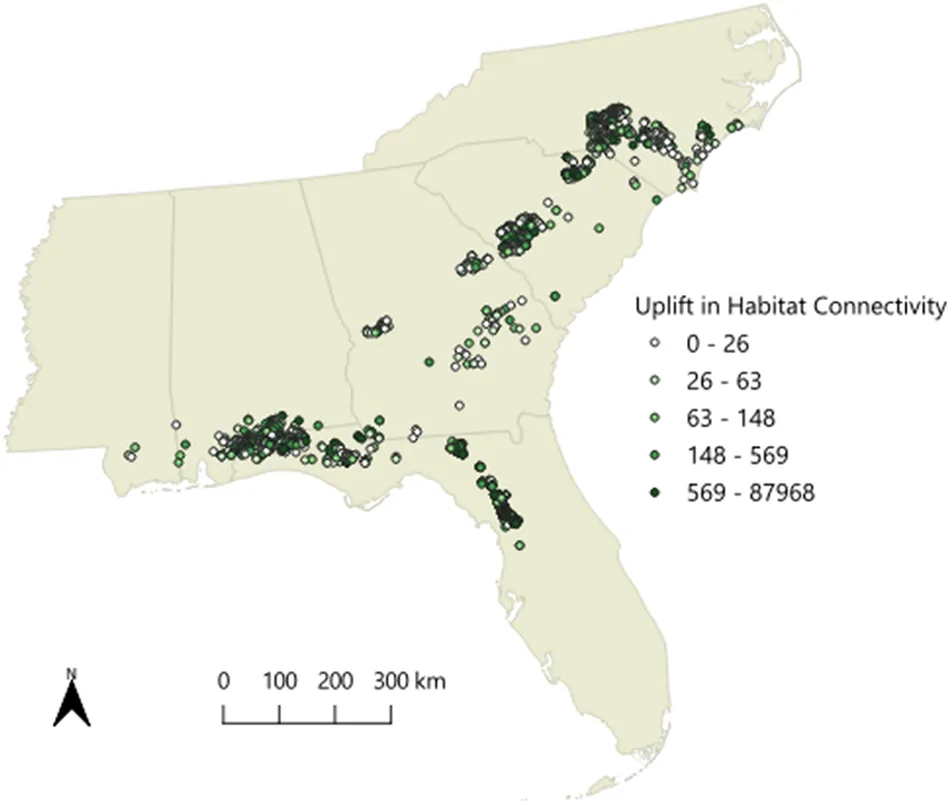
Map of potential opportunities for CRP longleaf pine in the South Atlantic Gulf

We identified 3103 agricultural fields that would be effective for habitat connectivity, are CRP eligible, and are suitable for longleaf pine restoration. Of these fields, 1283 were in Florida, 808 were in South Carolina, 796 were in North Carolina, 140 were in Georgia, 72 were in Alabama, and 4 were in Mississippi. From the ecological perspective, the Sandhills of Georgia, North Carolina and South Carolina had 1372 fields, the Southern Pine Plains and Hills of the Florida Panhandle had 686 fields, the Central Florida Ridges and Uplands had 442 fields, and the Carolina Flatwoods had 207 Fields. Other ecoregions of interest include the Atlantic Southern Loam Plains (GA, NC, SC), the Dougherty Plain (AL, FL, GA) and the Tallahassee Hills/Valdosta Limesink (FL and GA). The fields with the greatest uplift in habitat connectivity are spread throughout the region, mainly in central Florida, the Sandhills of North and South Carolina, and the Florida Panhandle (Fig. 3). See Tables S1 and S2 in the supplemental materials for the full descriptive statistics on the state-level and ecoregion-level results, respectively.

**Fig. 4:**
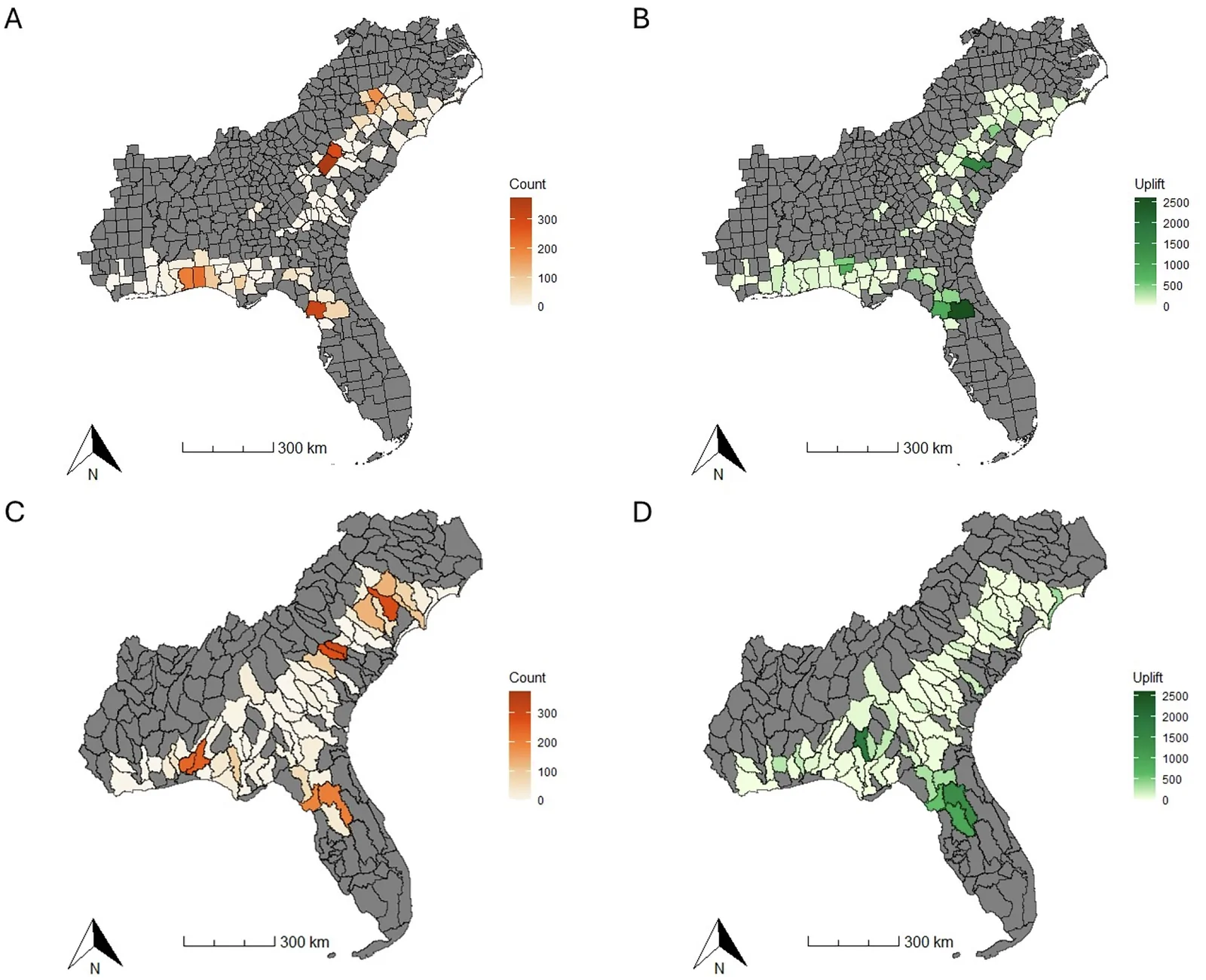
Maps of potential CRP longleaf pine opportunities by county and HUC 8 watersheds. Figure 4A displays the number (count) of opportunities at the county level, while Fig. 4C displays the number of opportunities at the HUC 8 watershed level. Figure 4B displays the median uplift in habitat connectivity for each county, while Fig. 4D displays the median uplift in habitat connectivity at the HUC 8 watershed level

There are 87 counties with at least one identified opportunity for CRP longleaf pine. Five counties have 200 or more opportunities for CRP longleaf pine: Aiken (376 fields) and Lexington (290 fields) counties in South Carolina, Levy (316 fields), Okaloosa (234 fields), and Santa Rosa (206 fields) counties in Florida (Fig. 4A). Among counties with more than one potential CRP field, the greatest uplift in habitat connectivity is observed in the central Florida counties of Marion and Levy, with median uplifts of 2,598.7 and 904.6, respectively (Fig. 4B). See Table S1 in the supplemental materials for the full descriptive statistics on the county-level results.

There are 63 watersheds with at least one identified opportunity for CRP longleaf pine. In South Carolina, the North Fork of the Edisto and the South Fork of the Edisto have the first and third most opportunities, with 309 and 279 fields, respectively. The Lumber watershed in North Carolina has the second most opportunities, with 289 fields (Fig. 4C). The Ocklawaha, Withlacoochee South and Waccasassa watersheds in Central Florida have high median uplift, with values of 1365.2, 963.4 and 610.6, respectively (Fig. 4D). See Table S3 in the supplemental materials for the full descriptive statistics on the HUC 8 watershed level results.

**Fig. 5:**
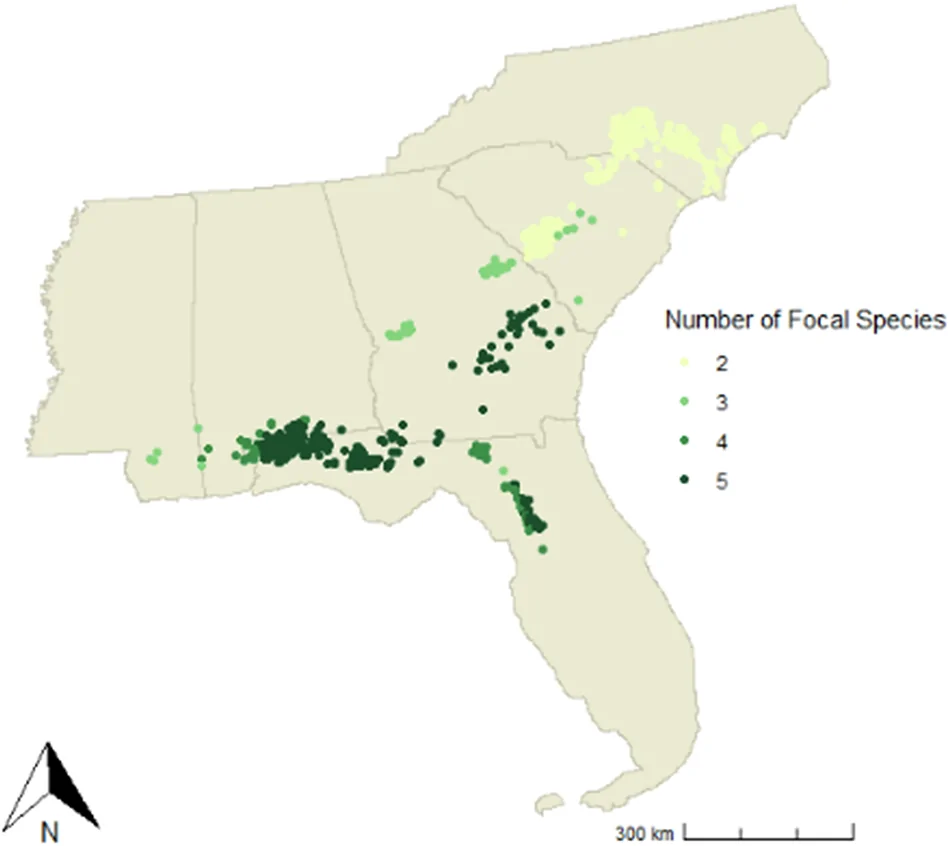
Map of the number of focal species whose ranges intersect with potential CRP longleaf pine opportunities

Regions where potential CRP longleaf pine opportunities are within the range of five focal species include the Loam Plains of Georgia, the eastern half of Central Florida, and the Florida Panhandle. Regions where potential CRP longleaf pine opportunities are within the range of four focal species include the Southern Pine Plains of Alabama and the western half of Central Florida. Potential CRP longleaf pine opportunities in the Sandhills of Georgia are within the range of three focal species. Potential CRP longleaf pine opportunities in the Sandhills of North Carolina and South Carolina, along with the Carolina Flatwoods, are within the range of two focal species (Fig. 5).

### Environmental Relationships

Of the five variables tested for correlation with uplift in habitat connectivity, none show a strong correlation ( > 0.7). Examining the linear regression model, all five independent variables were statistically significant, and the model itself was statistically significant, with an R-squared value of 0.46. The acreage of the field being converted to CRP longleaf pine had a positive relationship with uplift, as every one-percentage increase in field size resulted in a 1.4% increase in uplift. The percentage of existing longleaf pine in the 3 km buffer showed a negative relationship with uplift, as every one-percentage-point increase in longleaf pine resulted in a 3% decrease in uplift. The percentage of high-resistance pixels (road and developed land cover) in the 3 km buffer had a strong positive relationship with uplift, with every 1-unit increase in the percentage of high-resistance areas in the 3 km buffer resulting in a 7% increase in uplift. The PCI for longleaf pine showed a positive relationship with uplift: a 1-unit increase in PCI (longleaf pine patches becoming less isolated) was associated with a 3.9% increase in uplift. Landscape contagion had a positive relationship with uplift, as every 1-unit increase in contagion (land cover types in the landscape are becoming more clustered) resulted in a 2.9% increase in uplift (Table 3).

**Table 3:** Correlation and linear regression coefficients that display the relationships between uplift in habitat connectivity and different measures of the landscape

	Correlation	Linear Regression	Interpretation
Intercept	NA	-2.36***	NA
Acreage	0.68	1.40***	1.4% increase
Percentage of Longleaf Pine	−0.06	-0.03***	-3.1% decrease
Percentage of High Resistance Areas	0.14	0.06***	7.1% increase
Patch Cohesion Index (PCI)	0.01	0.04***	3.9% increase
Contagion	0.09	0.03***	2.9% increase
R-Squared	NA	0.46	NA
P-value	NA	0.0001	NA

***p < 0.001, **p < 0.01, *p < 0.05

The interpretation column represents the percent change in habitat connectivity uplift as a one unit/percent increase in the corresponding independent variable. With the exception of acreage (which is log transformed), interpretation values of the independent variables are derived from the exponential of the coefficients minus 1

### Effectiveness of the Spatial Prioritization Approach

**Fig. 6:**
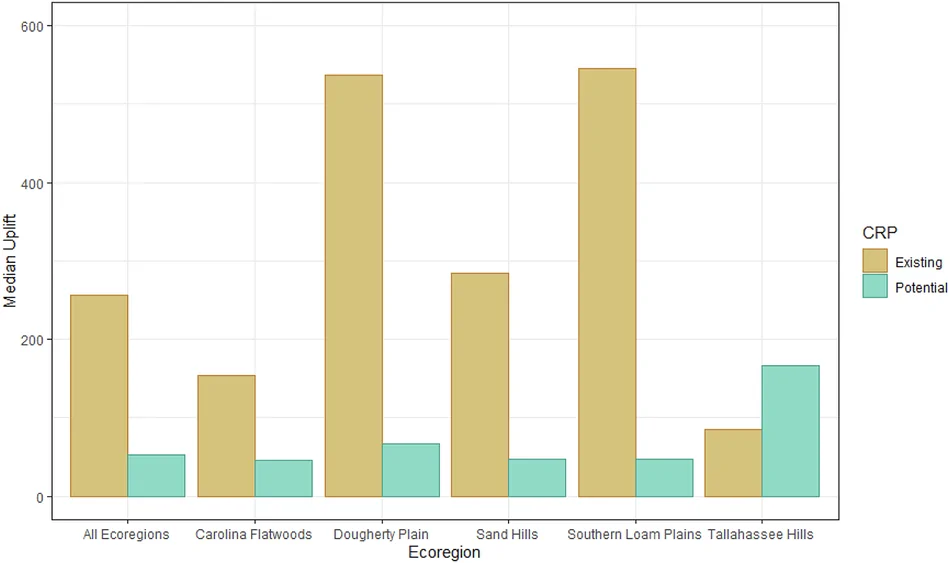
Median uplift values of the Mann-Whitney U tests for potential CRP longleaf opportunities, and existing CRP longleaf fields

For all regions, there is a statistically significant difference in median uplift values between the existing CRP longleaf pine fields and the potential CRP longleaf pine fields (Table S4 in the supplemental materials). For the dataset of the combined regions, the Atlantic Southern Loam Plains, the Dougherty Plain, the Carolina Flatwoods, and Sandhills, the median uplift is higher for existing CRP longleaf pine fields. In Tallahassee Hills/Valdosta Limesink, the median uplift is higher for potential CRP longleaf pine fields (Fig. 6). See Fig. S1 in the supplemental materials for the distribution of uplift values by ecoregion and see Fig. S2 in the supplemental materials for the distribution of uplift values for all five ecoregions combined.

## Discussion

Our results show the effectiveness of adopting a spatial prioritization approach for identifying future CRP longleaf opportunities to improve habitat connectivity. This, as confirmed by the coefficients in the linear regression model, indicates that our parameters were effective in identifying CRP longleaf pine opportunities and delineating the quality of different potential opportunities. The results of the Mann-Whitney U tests comparing the median uplift between the potential and existing CRP longleaf pine opportunities show that existing CRP longleaf pine opportunities have been successful in improving uplift in habitat connectivity. This is expected given that the USDA FSA and USDA NRCS frequently partner with organizations such as the Georgia Forestry Commission to identify prime opportunities for longleaf pine restoration through CRP (GFC, 2019; NRCS, 2020). It can be expected that on-the-ground knowledge and expertise of specific sites would be highly effective in improving habitat connectivity of longleaf pine-associated wildlife. The exception to this is the Tallahassee Hills/Valdosta Limesink ecoregion, where the potential CRP opportunities have a higher median uplift value than the existing CRP opportunities. This discrepancy could be attributed to the fact that Tallahassee Hills/Valdosta Limesink is in northcentral Florida, where CRP enrollment is low, suggesting that the CRP is currently underutilized in the region (FSA, 2025c). As CRP opportunities dwindle, data-informed approaches such as ours offer insights that, when integrated with on-the-ground knowledge, yield a robust framework for conservation planning.

The aggregated results shown in Fig. 4 provide conservation planners with insight into the resources required for CRP longleaf pine and how to allocate them. In counties and watersheds with many opportunities, conservation practitioners may choose to focus on a cluster of nearby opportunities to create a strong group of new longleaf pine patches. It is also important to consider the human dimensions of CRP enrollment, as landowners must voluntarily choose to enroll in the program. In areas where there are a lot of opportunities and/or resources and funding, conservation planners may decide to target landowners who are most willing to enroll in CRP, considering the high availability of CRP longleaf pine opportunities. On the other hand, areas with few opportunities or limited resources may choose to focus on targeted outreach in high-uplift fields to maximize conservation outcomes. Another dimension of the aggregated results is the opportunity to couple habitat connectivity with other ecosystem services, such as soil erosion and water quality. This is especially useful at watershed scales, as it enables conservation planners to integrate habitat connectivity metrics into a watershed modeling framework. Given the limited resources available to CRP, targeting multiple environmental goals within a single CRP field offers a cost-effective approach to conservation planning.

The intersection of species ranges with potential CRP longleaf pine opportunities, as shown in Fig. 5, provides an additional dimension for considering parameters of spatial prioritization in conservation planning. The inclusion of species range overlap provides enhanced value to CRP longleaf pine opportunities in the Georgia Sandhills. Opportunities in the Georgia Sandhills are low in uplift and not in close proximity to each other (limiting the opportunity for compounding uplift through multiple fields), but the intersection of five focal species with CRP longleaf pine opportunities means that the Georgia Sandhills can provide a wide range of connectivity benefits.

While opportunities are locationally clustered, Fig. 3 shows that opportunities are not clustered from an uplift perspective. The coefficients of the linear regression model indicate key relationships between uplift in habitat connectivity and environmental characteristics of the field and the surrounding landscape. The positive relationship between uplift and the size of the potential CRP field indicates that larger fields should be prioritized for CRP longleaf pine. As larger fields are converted to longleaf pine, more high-resistance land cover/pixels are removed from the landscape and replaced with low-resistance land cover/pixels, as they provide more coverage for longleaf pine-associated species to move through the landscape at less cost. Measures of landscape composition have strong but opposing influences on uplift in habitat connectivity. The relationship between landscape configuration and uplift suggests the importance of the existing longleaf pine and other land cover types for improving habitat connectivity. Both variables (PCI of longleaf pine patches and contagion of the landscape) indicate that when existing longleaf pine patches in the local landscape are aggregated and/or clustered, the benefit to habitat connectivity is greater compared to isolated patches. This makes sense from an ecological perspective—placing new longleaf pine near existing patches provides strong corridors for wildlife to move between existing stands and reduces the number of higher-resistance pixels that an individual would have to move through.

Our results are in line with previous research that analyzes the effect of the CRP on landscape structure. Tanner and Fuhlendorf (2018) found that in Oklahoma, the CRP helped improve habitat connectivity and maintained the complexity of grassland landscapes. Similarly, Park and Egbert (2008) found that in southwest Kansas, the CRP assisted in the reversal of habitat fragmentation. While these results are centered on grassland landscapes in the Great Plains of the United States, these results, paired with ours, show the ability of the CRP to improve habitat connectivity across a suite of different landscapes.

Additionally, our spatial prioritization approach mostly aligns with FNAI’s Longleaf Sustainability Analysis Priority Areas of Restoration, with both our approach and FNAI’s approach identifying the Florida Panhandle, the Georgia Sandhills, the Carolina Sandhills, and the Carolina Flatwoods as high-priority restoration areas. Our results place a greater prioritization on longleaf restoration in the western portion of the Central Florida Ridges and Uplands, and in the Southern Loam Plains of Georgia. Our results do not capture the FNAI’s high prioritization of central Alabama and the eastern portion of the Central Florida Ridges and Uplands (FNAI and UF CLCP, 2023). Our results also align with studies that focus on the regional prioritization of longleaf pine. Murray et al. (2023) identified longleaf pine restoration opportunities in the South Carolina Sandhills Wiregrass Gap. Our results and Murray et al. (2023) both identified high-priority longleaf pine restoration opportunities south of Lake Murray near the North Fork Edisto River, and southwest of the Carolina National Wildlife Refuge. Hoctor et al. (2025) identified conservation priority areas for protecting Florida’s biodiversity and ecosystem services. Our approach found longleaf pine restoration opportunities that intersect with areas in the Florida Panhandle that Hoctor et al. (2025) identified as high priority for conservation.

## Conclusion

Despite having a lower median uplift than the existing CRP longleaf pine fields, our approach was effective for identifying new potential CRP longleaf pine opportunities in the South Atlantic Gulf. Our results identify geographic clusters of CRP-related longleaf pine opportunities in the Central Florida Ridges and Uplands, the Sandhills of North Carolina and South Carolina, and the hills and plains of southern Alabama and the Florida Panhandle. The Central Florida Ridges and Uplands offer the greatest opportunity to improve habitat connectivity through CRP longleaf pine, given the high quantity and quality of potential sites. Furthermore, CRP longleaf pine opportunities in the Central Florida Ridges and Uplands intersect with the ranges of five focal species, meaning that they can provide benefits to a wide range of longleaf pine-associated species. The other three areas have a mix of both low and high connectivity opportunities, dependent on the field’s location in the landscape. Results of the linear regression model indicate the importance of site-specific traits (field acreage) and composition and configuration of the local landscape for prioritization.

Using an omnidirectional cost surface approach, our study builds on existing research assessing CRP’s effectiveness at improving habitat connectivity for wildlife in two unique ways. First, our study is focused on the southeastern US, an understudied region in regard to CRP-related research. Second, because of the focus on longleaf pine ecosystems, our results provide a generalized connectivity framework (i.e., not developed for a specific wildlife species) for measuring habitat connectivity among longleaf pine-associated species. We would like to note that we use generalized connectivity to provide a streamlined approach to understanding the CRP’s effect on habitat connectivity. Species-specific connectivity is dependent on the species’ dispersal and movement ability. Beyond the literature, our research provides a spatial planning framework for conservation practitioners to adopt and adapt, thereby meeting and expanding conservation goals related to longleaf pine. Our results indicate the effectiveness of CRP in improving habitat connectivity in the Southeast, an area that has not been studied before. We would like to emphasize that our work specifically assesses the effectiveness of the CRP for improving habitat connectivity and should not be used as a framework for evaluating the effectiveness of the CRP for providing or improving wildlife habitat. We would also like to emphasize that our results are focused solely on identifying opportunities for improving the connectivity of the longleaf pine landscape and its effect on improving habitat connectivity. Our research does not cover the human dimensions of CRP adoption, which includes the willingness to enroll in the CRP and the feasibility of on-the-ground management for implementing longleaf pine. Future directions for this research include integrating a measure of a landowner’s willingness to enroll in the CRP to create a more holistic priority metric, allowing conservation planners to consider both the environmental and human dimensions of the CRP. It is also important to note that uplift represents the best-case scenario in improving connectivity and may not necessarily represent the “on the ground” adoption and management of CRP longleaf pine.

While our spatial prioritization approach was developed for longleaf pine restoration through CRP in the southeastern US, our methods are transferable to similar land retirement programs in other countries, such as China’s Grain for Green Program, where cropland is converted into forests, grasslands or shrublands (Deng et al., 2014). Our approach can also be applied to the afforestation component of the European Union’s (EU) Biodiversity Strategy for 2030, which aims to support biodiversity-friendly afforestation as part of the EU’s pledge to plant 3 billion additional trees by 2030. The abandonment of agricultural land in Europe presents an opportunity for afforestation and has been a focus of afforestation efforts over the past few decades. However, previous afforestation efforts have resulted in the loss of non-forest biodiversity and failure due to poor site conditions (European Commission, 2023). Our approach can be tailored to the EU’s biodiversity-friendly afforestation goals, as our approach focuses on identifying land where existing forest stands are present in the nearby landscape, selecting land that will help biodiversity by increasing habitat connectivity.

## Supplementary information

LLPConnectivity_SI_R2

LLPConnectivity_SI_Tables_R2

## Data Availability

Data are not available due to privacy concerns. However, interested parties can contact the first author to explore suitable ways to share the data, considering the limitations of the existing agreements.
