# Supplementary material for: A Spatial Prioritization Approach for Identifying Future Conservation Reserve Program Opportunities to Enhance Longleaf Pine (Pinus palustris) Ecosystem Connectivity
Source: Environ Manage. 2026 Sep 3;76(9):303. doi: 10.1007/s00267-026-02593-z (PMC13541870; doi:10.1007/s00267-026-02593-z)
Supplement: Supplementary file 1 [file 267_2026_2593_MOESM1_ESM.docx]

**A Spatial Prioritization Approach for Identifying Future Conservation Reserve Program Opportunities to Enhance Longleaf Pine (*Pinus palustris*) Ecosystem Connectivity**

Benjamin C. Townsend ^a^*, Nathan P. Nibbelink ^a^, and Puneet Dwivedi ^b^

^a^Warnell School of Forestry and Natural Resources, University of Georgia, Athens, Georgia, 30602, United States of America; ^b^Department of Forestry and Environmental Conservation, Clemson University, Clemson, South Carolina, 29634, United States of America

*Benjamin C Townsend, Warnell School of Forestry and Natural Resources, University of Georgia, 180 E Green St, Athens, Georgia, 30602, United States of America. Email: [btownsend@uga.edu](mailto:btownsend@uga.edu)

**Supplemental Tables**

*Table S1: Descriptive statistics for uplift in connectivity at the state and county Levels. Entries in bold are for state-level results. Counties that are listed below a state belong to that state. “Count” displays the number of potential CRP fields in the specified county/state. “MeanUplift” measures the mean uplift in habitat connectivity for potential CRP in the specified county/state. “MedUplift” measures the median uplift in habitat connectivity for potential CRP in the specified county/state. “SdUplift” measures the standard deviation in uplift in habitat connectivity for potential CRP in the specified county/state. “MinUplift” measures the minimum uplift in habitat connectivity for potential CRP in the specified county/state. “MaxUplift” measures the maximum uplift in habitat connectivity for potential CRP in the specified county/state.*

*Table S2: Descriptive statistics for uplift in connectivity at the Level 4 Ecoregion scale. “Count” displays the number of potential CRP fields in the specified ecoregion. “MeanUplift” measures the mean uplift in habitat connectivity for potential CRP in the specified ecoregion. “MedUplift” measures the median uplift in habitat connectivity for potential CRP in the specified ecoregion. “SdUplift” measures the standard deviation in uplift in habitat connectivity for potential CRP in the specified ecoregion. “MinUplift” measures the minimum uplift in habitat connectivity for potential CRP in the specified ecoregion. “MaxUplift” measures the maximum uplift in habitat connectivity for potential CRP in the specified ecoregion.*

*Table S3: Descriptive statistics for uplift in connectivity at the HUC8 Watershed Level. “Count” displays the number of potential CRP fields in the specified watershed. “MeanUplift” measures the mean uplift in habitat connectivity for potential CRP in the specified watershed. “MedUplift” measures the median uplift in habitat connectivity for potential CRP in the specified watershed. “SdUplift” measures the standard deviation in uplift in habitat connectivity for potential CRP in the specified watershed. “MinUplift” measures the minimum uplift in habitat connectivity for potential CRP in the specified watershed. “MaxUplift” measures the maximum uplift in habitat connectivity for potential CRP in the specified watershed.*

*Table S4: Results of the Mann-Whitney U tests between the median uplift of potential CRP opportunities and the median uplift of existing CRP opportunities.*

Note: The supplemental tables are included in separate Excel files due to the size and length of the tables. A caption is included for each table in this document.

**Supplemental Figures**

***Fig. S1:*** Distribution of uplift values between existing and potential CRP opportunities, split by Ecoregion. NOTE: The x limit was capped at 5000 to better display the difference in distribution between the two groups. As a result, 49 high outliers were removed from the plot.

***Fig. S2:*** Distribution of uplift values for all 5 ecoregions between existing and potential CRP opportunities. NOTE: The x limit was capped at 5000 to better display the difference in distribution between the two groups. As a result, 49 high outliers were removed from the plot.

***Fig. S3*** Map of CRP eligible lands up to 2025. Pixel values represent the percentage of the land within the 3-kilometer pixel that is CRP eligible.

***Fig. S4*** Map of existing longleaf pine. Pixel values represent the percentage of the land within the 3-kilometer pixel that is covered by longleaf pine.

***Fig. S5*** Map of land suitable for longleaf pine. Pixel values represent the percentage of the land within the 3-kilometer pixel that is suitable for longleaf pine.
